# Supplementary material for: Infant Feeding Choices during the First Post-Natal Months and Anthropometry at Age Seven Years: Follow-Up of a Randomized Clinical Trial
Source: Nutrients. 2022 Sep 21;14(19):3900. doi: 10.3390/nu14193900 (PMC9572598; doi:10.3390/nu14193900)
Supplement: Supplementary file 1 [file nutrients-14-03900-s001.zip › nutrients-1894576-supplementary.pdf]

Supplemental Table S1 Comparison of seven-year follow-up participants and non participants, intent-to-treat population.

	participants	non participants	*p*-value
Male sex (%)	54.0	43.2	0.093
Maternal education Basic/Additional/Tertiary (%)	1.9/60.9/37.3	9.5/53.7/36.8	0.019
Mother smoked during pregnancy (%)	20.5	32.6	0.030
Age of mother at delivery (years, mean±SD)	31.7±4.8	29.7±5.6	0.004

Continuous variables tested with Student´s t-test (Satterthwaite), categorical data tested with Chi-Square test

Supplemental Table S2. Anthropometry and body composition at seven years of age follow-up (mean±SD), per protocol population.

	**Formula groups**				**Breastfed group**		
	**Intervention (*n*=32)**	**Control (*n*=20)**	**IF vs. CF[1]**	**Estimated difference between IF and CF**	**(*n*=36)**	**IF vs. BF[1]**	**CF vs. BF[1]**
Weight (kg)	26.7±4.8	25.4±3.3	0.267	0.99 [-1.57;3.54][2]	26.4±5.2	0.835	0.380
Weight-for-age (z-score)	0.95±1.06	0.68±0.92	0.347	0.22 [-0.38;0.82][2]	0.88±1.15	0.813	0.479
Height (cm)	128.9±5.0	128.2±4.1	0.597	0.56 [-2.18;3.30][2]	129.0±5.1	0.928	0.534
Height-for-age (z-score)	1.41±0.93	1.30±0.77	0.648	0.12 [-0.39;0.63][2]	1.42±0.94	0.951	0.598
Head circumference (cm)	52.8±1.9	52.5±1.6	0.512	0.22 [-0.71;1.16][2]	52.7±1.5	0.766	0.653
BMI (kg/m²)	16.0±2.5	15.4±1.4	0.265	0.46 [-0.74;1.65][2]	15.8±2.7	0.779	0.436
BMI-for-age (z-score)	0.15±1.28	-0.14±1.09	0.390	0.22 [-0.44;0.88][2]	0.00±1.48	0.671	0.678
Body fat from BIA (%)	14.9±7.6	14.6±5.4	0.980	0.35 [-3.67;4.38][3]	12.8±6.9	0.231	0.215
Triceps (mm)	11.6±4.6	10.4±3.5	0.314	1.40 [-0.90;3.70][3]	11.2±3.9	0.750	0.428
Subscapular (mm)	7.4±3.8	6.4±1.5	0.196	1.05 [-0.76; 2.86][3]	7.6±3.9	0.880	0.133
Body fat from skinfolds (%) [1]	17.7±6.0	16.0±4.0	0.231	1.91 [-1.08;4.90][3]	17.6±5.6	0.936	0.229

Mean ± SD. [1] p-values from Student´s t-test (Satterthwaite), significance at $p<0.05$ [2] Slaughter et al. 1988 [2] Derived from ANCOVA adjusted for age, maternal age at 7-year-follow-up, sex, smoking mother at 7-year-follow-up, value at age 1 month: : mean [95% CI] [3] Derived from ANCOVA adjusted for age, maternal age at 7-year-follow-up, sex, smoking mother at 7-year-follow up: mean [95% CI]

Supplemental Table S3. Absolute change of weight, length and BMI z-scores (mean±SD) at 7 years of life in comparison to month one, month four and four years (IF = interventional formula, CF = control formula, BF = breast fed), intent-to-treat population.

	n	IF	*n*	CF	IF vs. CF[a]	*n*	BF	IF vs. BF[a]	CF cv. BF[a]
**Change in weight-for-age z-scores**									
7 years – 1 month	54	1.32±1.14	50	1.15±1.02	0.428	57	0.87±1.08	0.038	0.177
7 years – 4 months	54	0.72±1.02	50	0.99±0.88	0.149	57	0.83±1.08	0.591	0.391
7 years – 4 years	52	0.32±0.69	48	0.31±0.67	0.902	57	0.16±0.61	0.185	0.232
**Change in height-for-age z-scores**									
7 years – 1 month	54	1.16±1.08	50	1.09±1.01	0.731	57	0.69±1.02	0.021	0.045
7 years – 4 months	54	0.80±0.96	50	1.00±0.82	0.264	57	0.85±0.82	0.771	0.360
7 years – 4 years	52	0.35±0.57	48	0.41±0.56	0.547	57	0.45±0.51	0.326	0.747
**Change in BMI-for-age z-scores**									
7 years – 1 month	54	0.89±1.35	50	0.70±1.15	0.434	57	0.57±1.45	0.222	0.596
7 years – 4 months	54	0.32±1.38	50	0.52±1.16	0.437	57	0.37±1.32	0.836	0.555
7 years – 4 years	52	0.18±0.94	48	0.12±1.00	0.744	57	-0.17±0.89	0.046	0.120

[a] *p*-values from Student´s t-test (Satterthwaite), significance at $p<0.05$
